# Cannabidiol (CBD) and Other Cannabinoids as a Promising Alternative Antibacterial Agent—Pilot Study on *Enterococcus faecalis* and *Enterococcus faecium* Clinical Strains

**DOI:** 10.3390/molecules31010144

**Published:** 2026-01-01

**Authors:** Zuzanna Kraszewska, Katarzyna Grudlewska-Buda, Kacper Wnuk, Ewa Wałecka-Zacharska, Eugenia Gospodarek-Komkowska, Krzysztof Skowron

**Affiliations:** 1 Department of Microbiology, Ludwik Rydygier Collegium Medium in Bydgoszcz, Nicolaus Copernicus University, 87-100 Toruń, Poland; z.kraszewska@cm.umk.pl (Z.K.); k.grudlewska@cm.umk.pl (K.G.-B.); gospodareke@cm.umk.pl (E.G.-K.); 2 Clinical Microbiology Division, Antoni Jurasz University Hospital No. 1 in Bydgoszcz, 85-094 Bydgoszcz, Poland; 3 Department of Theoretical Foundations of Biomedical Sciences and Medical Computer Science, Ludwik Rydygier Collegium Medium in Bydgoszcz, Nicolaus Copernicus University, 87-100 Toruń, Poland; kacper.wnuk@cm.umk.pl; 4 Department of Food Hygiene and Consumer Health, Wrocław University of Environmental and Life Sciences, 50-375 Wrocław, Poland; ewa.walecka@upwr.edu.pl

**Keywords:** hemp oils, cannabidiol, *Enterococcus*, cannabinoids, antibacterial

## Abstract

Gram-positive cocci of the *Enterococcus* genus, despite their prevalence in the environment and the microbiota of healthy people, have become a serious threat in hospitals as opportunistic pathogens. These bacteria have many virulence factors and intrinsic resistance to existing drugs, which significantly narrows the group of effective antimicrobials. Due to the spread of Multi-Drug-Resistant (MDR) strains, there is a need to search for new substances as potential antibiotics. Our work aimed to evaluate the antimicrobial effect of commercially available products (five oils containing cannabidiol (CBD) and its derivatives and one 99% CBD product in the form of crystals) on 20 clinical strains of *E. faecalis* and *E. faecium.* We determined the Minimal Inhibitory Concentration (MIC) of CBD oils using the microdilution method in Mueller–Hinton broth (MHB). The CBD displayed antibacterial properties against all tested *Enterococcus* spp. strains (MIC ≤ 1 μg/mL). The higher concentration of CBD resulted in a larger antibacterial effect. The obtained MICs of pure CBD and CBD crystals were statistically lower (W = 97, *p* < 0.001) for *E. feacium* than *E. faecalis*. This work confirms the antibacterial activity of CBD on *Enterococcus* spp., providing a solid basis for further research that can help identify new therapeutic options and gain a deeper understanding of the CBD mechanism of action.

## 1. Introduction

*Enterococcus* spp. are facultative anaerobic Gram-positive cocci frequently isolated from the environment, including soil, water, and sewage. These bacteria are also a component of the human and animal gastrointestinal natural microbiota [1]. Two species, *Enterococcus faecalis* and *Enterococcus faecium*, isolated mainly from hospitalized patients, can cause urinary tract infections [2], endocarditis [3], wound infections, or even sepsis [4]. According to recent European Centre for Disease Prevention and Control (ECDC) reports, *Enterococcus* spp. are the second leading etiological agent of urinary tract infections (UTI) and bloodstream infections in intensive care units (ICU) and are responsible for 15.1% of surgical site infections (SSI) in Europe [5,6]. The pathogenic potential of *Enterococcus* spp. is closely related to the cell surface components (polysaccharide capsule, adhesins, pili, and aggregating substance) as well as the ability to produce bacteriocins and enzymes (hemolysin, cytolysin, gelatinase, serine protease) [7]. Many of these virulence factors affect the formation and maintenance of a biofilm structure, which promotes adhesion to catheters, prostheses, and artificial heart valves, contributing to the persistence of antibiotic-resistant infections [8,9].

*Enterococcus* spp. have multiple intrinsic mechanisms resulting in predicted resistance to cephalosporins, meropenem, macrolides, and sulfonamides [10]. Additionally, the natural expression of a penicillin-binding protein (pbp5) and low permeability of the enterococcal membrane contribute to high resistance to beta-lactams and aminoglycosides [11]. Of the highest importance is the resistance to glycopeptides. Vancomycin resistance is associated with the presence of genes (*vanA*, *vanB*, *vanC*, *vanD*) located on mobile elements of the *Enterococcus* spp. genome, called pathogenicity islands (PAI) [12]. In 2024, the percentage of infectious vancomycin-resistant *Enterococcus* spp. (VRE) exceeded 25% in 10 European countries [13]. In the case of strains that are also resistant to gentamicin (Glycopeptides-resistant *Enterococcus*, GRE), therapeutic options are often very limited [14,15,16,17].

Due to the rapid spread of MDR strains, there is a need to discover new antimicrobial substances. The alternative can be known but forgotten organic natural compounds. In recent years, there has been intense interest in substances of plant origin called cannabinoids. Out of 125 substances extracted from the hemp plant *Cannabis sativa*, a few have therapeutic activity. In 2017, the Expert Committee on Drug Dependence (ECDD) officially announced that CBD in its pure form is non-addictive and harmless to health [15] and can be used in the treatment of numerous non-infectious diseases [16]. Most importantly, it has bactericidal potential against Gram-positive bacteria and can selectively kill Gram-negative bacteria [17]. Since then, several scientific reports with promising results on the antibacterial effect of CBD against various Gram-positive bacteria have appeared [18,19,20]. However, studies focused on *Enterococcus* spp. are rare or limited to a small number of strains.

Since there is a necessity to seek new therapeutic options against increasingly resistant *Enterococcus* spp. and CBD-containing oils, our work aimed to assess the antibacterial activity of commercial products containing CBD and other cannabinoids.

## 2. Results

### 2.1. Identification of Strains and the Assessment of Antimicrobial Susceptibility to Selected Antibiotics

For every strain, the identification parameter called the Score Value (SV) was ≥2.00, which means a highly probable identification of the species (Table 1). The sensitivity to tested antibiotics was more frequently reported among *E. faecalis* strains than *E. faecium* strains. There were four *E. faecalis* strains susceptible to all the tested antibiotics. All of the *E. faecium* strains were resistant to beta-lactams (ampicillin and imipenem), while six of the *E. faecalis* strains were susceptible to those antibiotics. There were eight strains (seven *E. faecium* isolates and one *E. faecalis*, 40%) resistant to vancomycin (VRE). Four strains (three *E. faecium* and one *E. faecalis*, 20.00%) were resistant to both vancomycin and teicoplanin (GRE). Four strains (two *E. faecium* and two *E. faecalis*, 20.00%) were Linezolid-resistant Enterococcus (LRE).

For vancomycin, there was an accordance between the interpretation of the MIC values and the disc-diffusion method. In contrast, two out of four strains that were resistant to teicoplanin based on the disk-diffusion method remained susceptible to this antibiotic, according to the interpretation of the MIC value presented in EUCAST [21].

### 2.2. Determination of Pure Reference Cannabidiol and CBD Crystals Minimum Inhibitory Concentration

The determined pure cannabidiol MIC values (identical in triplicate) for all 11 *E. faecium* isolates were ≤0.5 and for nine *E. faecalis* ≤ 1. For each of the tested strains, the MIC values of the CBD crystals were equal to the MIC values of the reference pure substance except for strain 15 (*E. faecalis*). A non-parametric Wilcoxon rank-sum test was performed to compare the distributions of the MIC values of *E. faecalis* and *E. faecium* (W = 97, *p* < 0.001). The details are presented in Table 2.

### 2.3. Determination of Cannabinoid Oils’ Minimum Inhibitory Concentration

All the MIC values obtained in triplicate were identical. Among the *E. faecium* strains, the MIC values for CBD 20% GOLD oil were ≤0.5 μg/mL, and in 63.64% (7 out of 11 isolates), they were equal to the MIC values for the reference substance (*p* < 0.001). The CBD 20% GOLD oil MIC values for *E. faecalis* ranged from 1 to 4 μg/mL, and for eight out of nine tested isolates (88.89%), they were higher compared to the reference substance (*p* < 0.001). The MIC values of the CBD oil 20% RAW for *E. faecium* strains ranged from 0.25 to 1 μg/mL, and for *E. faecalis* strains, they ranged from 0.5 to 8 μg/mL. The MIC values of CBD 20% GOLD and CBD 20% RAW oils were equal for 9 out of 20 tested strains (45.00%). The MIC of the CBN + CBD 10% RAW oil for *E. faecium* strains was ≤0.5 μg/mL, and for the majority of *E. faecalis* strains (88.89%), it was >16 μg/mL. An ordinal logistic mixed-effect model was fitted to assess the impact of the species and oil type (including pure CBD) on the MIC values. The analysis revealed statistically significant differences in the MIC values between the two species (χ2 = 31.04, *p* < 0.001) and different cannabinoid oils used (χ2 = 89.64, *p* < 0.001). Detailed MIC values for the tested cannabinoid oils are presented in Table 3.

## 3. Discussion

Although the first reports on the antibacterial properties of cannabinoids date back to the 1950s [22,23], in recent years, studies on the impact of CBD against various bacteria have gained popularity. While CBD showed weak antibacterial properties against Gram-negative bacteria [24], Pisanti et. al. [25] demonstrated its activity against methicillin-resistant *Staphylococcus aureus* (MRSA) strains. This discovery was reported in the ECDD Cannabidiol Pre-Review report on the potential therapeutic use of this substance in infections [26]. An official ECDD Cannabidiol Critical Review Report in 2018 confirmed this information, indicating that the research is only preliminary or at the pre-clinical stage [16].

So far, the effect of CBD has been assessed on single *Enterococcus* spp. strains. Blaskovich [17] et al. determined the antimicrobial properties of CBD for three *E. faecalis* (MIC 1–4) and four *E. faecium* strains (MIC = 0.5–1), whereas Abichabki [27] et al. studied six *Enterococcus* spp. strains (MIC = 2–4). In contrast, our work evaluates the MIC of CBD and mixed cannabinoid preparations on a significantly larger number of *Enterococcus* spp. strains (20 isolates), making the obtained results more reliable. Additionally, all of our strains were of clinical origin. To our knowledge, this is the first work to compare the antibacterial effects of different preparations containing CBD and other cannabinoids, which may guide future studies on these compounds.

The first purpose of this study was to compare the pure CBD substance’s (Phytolab^®^) and 99% CBD crystals’ (Enecta^®^) activity against 20 selected clinical strains of *E. faecalis* and *E. faecium*. We found that for 19 out of 20 (95.0%) strains, the MIC values [μg/mL] for both products were identical (MIC ≤ 1). For only one of the *E. faecalis* strain, the MIC value of CBD in the form of crystals was two times lower (MIC = 0.5) than the MIC value of the pure substance (MIC = 1). To analyze the effect of the CBD products on *Enterococcus* spp., a non-parametric aligned rank transform (ART) procedure was conducted. The analysis revealed no significant difference between pure CBD and CBD crystals (*p* < 0.001). We plan to expand the group of studied strains to obtain more valuable results, which could increase interest and lower costs in this type of research.

We observed that the MICs of pure CBD and CBD crystals were lower for *E. faecium* than for *E. faecalis* (W = 97, *p* < 0.001), which may be explained by differences in the metabolic processes of these species [28]. Currently, the mechanism of action of cannabinoids, including CBD, on bacterial cells is not fully described. Zeng et al. studied the mechanism of CBD’s antibacterial action on various Gram-positive bacteria, including *E. faecium*. They suggested that CBD disrupts cell membranes by altering the bacterial proteomic and metabolic profile, impeding phosphorylation, and inhibiting amino acid biosynthesis [29]. We believe that examining the metabolic pathways and global transcriptome of both species could help better understand the mechanism of CBD action. We applied an ordinal logistic mixed-effect model to determine whether the species and cannabinoid products (including pure CBD) significantly affected the MIC values. The analysis revealed that the inhibitory effect depended on the species (χ2 = 31.04, *p* < 0.001) and cannabinoid product used (χ2 = 89.64, *p* < 0.001). Moreover, a significant two-way interaction (χ2 = 71.29, *p* < 0.001) was observed, suggesting that the differences between cannabinoid oils (or pure CBD) were dependent on the species, which may be due to the various mechanisms of cannabinoid metabolism by *E. faecalis* and *E. faecium*. Post hoc analyses with Holm’s correction demonstrated distinct patterns between cannabinoid oils and pure CBD. For *E. faecalis*, a consistent increase in the MIC values was observed, with all materials showing significantly higher log-odds estimates relative to the reference substance (*p* < 0.001). However, for *E. faecium*, this pattern was less distinct; significant increases in log-odds relative to pure CBD were found only for CBDA + CBD 5% RAW (difference in log-odds = 7.70, *p* < 0.001) and CBG 10% RAW (difference in log-odds = 4.93, *p* < 0.001), while the other oils did not differ significantly from the pure CBD. This analysis allowed us to conclude that CBD 20% GOLD, CBD 20% RAW, and CBN + CBD 10% RAW preparations had the highest antibacterial properties against *Enterococcus* spp. as compared to the reference substance and are more effective against *E. faecium* than *E. faecalis*. A higher concentration of CBD in oils resulted in a larger antibacterial effect of the selected preparations.

It should be noted that the observed differences in the MIC of oil products compared to the reference substance may also be influenced by their physicochemical properties. Hazekamp A. indicated that additional components present in unpurified cannabinoid preparations can spontaneously convert into other forms [30]. In addition, the oily form of the product itself can affect the MIC values, as it can partially encapsulate the active substance in an aqueous solution with a low ethanol content (5%). Iseppi et al. determined the MIC values of 17 hemp oils and pure CBD for many bacterial isolates of food origin, including seven *Enterococcus* spp. strains [31]. The MIC values of the hemp oils ranged between 0.25 and 32 [μg/mL], while for pure CBD, they ranged from 1 to 4 [μg/mL]. We also noted a large discrepancy in the MIC range [from 0.125 to >16 [μg/Ml]] for oils containing higher concentrations of admixtures (>40.0%) of other than CBD components (CBN, CBG, CBDA, terpenes). This leads to difficulties in indicating the main antibactericidal active substance in this preparation. We found no articles comparing the MIC values of known cannabinoids against *E. faecium* and *E. faecalis*, which confirms that the presented pilot studies provide new observations.

We would like to emphasize that the presented results are pilot studies that constitute the basis for further research, including the expansion of the study group, analysis of the genetic background, and the effect of CBD on biofilm formation by *Enterococcus* spp. At this stage, the obtained results may help other researchers plan and select preparations based on the described methodology.

## 4. Materials and Methods

We selected 20 clinical *E. faecalis* and *E. faecium* strains isolated from urinary tract infections from patients of Dr. Antoni Jurasz University Hospital No. 1 in Bydgoszcz, Poland, in 2023–2024. Colonies grown from clinical cultures were streaked onto Columbia Agar (BioMaxima, Lublin, Poland). After 24 h of incubation at 37 °C, the isolates were preserved in nutrient broth (Merck, Darmstadt, Germany), supplemented with 15% glycerol (Avantor Sciences, Gliwice, Poland), and stored at −80 °C until use.

The inclusion criteria were isolated strains from different patients of urinary tract infection origin, species *E. faecalis* or *E. faecium*, and phenotypic diversity in antimicrobial susceptibility pattern. We selected 10 isolates with resistance mechanisms (GRE, VRE, LRE) and 10 isolates susceptible to glycopeptides and linezolid. Detailed information about the tested strains is provided in Table 4.

### 4.1. Identification of Strains

Strain identification was performed by MALDI-TOF MS (Matrix Assisted Laser Desorption/Ionization–Time of Flight Mass Spectrometry), using the MALDI BioTyper Microflex mass spectrometer (Bruker, Billerica, MA, USA), according to the manufacturer’s procedural recommendations. The credibility of the obtained results was determined on the basis of a parameter called the “Score Value”, which was interpreted based on the system’s guidelines (a score ≥2.00 means a highly probable result).

### 4.2. Assessment of Antimicrobial Susceptibility to Selected Antibiotics

The susceptibility of *Enterococcus* spp. strains to antibiotics was assessed using the Kirby–Bauer disc diffusion method on Mueller–Hinton II Agar (MHA, Becton Dickinson, Franklin Lakes, NJ, USA), according to the EUCAST (European Committee for Microbial Susceptibility Testing) recommendations [32]. The test was performed using ampicillin (2 μg), imipenem (10 μg), vancomycin (5 μg), teicoplanin (30 μg), and linezolid (10 μg) discs (OXOID, Basingstoke, UK). The zone sizes around the discs were interpreted using EUCAST ver. 13.0 breakpoint tables after incubation [21]. The reference strain *E. faecalis* ATCC^®^ 29212™ from the American Type Culture Collection (ATCC^®^, Gaithersburg, MD, USA) was used as a control.

Additionally, the susceptibility of the tested strains to vancomycin and teicoplanin was assessed by determining the MIC values, according to the EUCAST recommendations [21].

### 4.3. Assessment of the Minimum Inhibitory Concentrations of Cannabinoid Products

To evaluate the antibacterial properties of CBD and products containing CBD in combination with other cannabinoids, we selected 6 commercial products (one in the form of CBD crystals with an admixture of 1% terpenes and 5 oils containing cannabinoids and terpenes in various proportions). Two forms of oils were applied: purified from chlorophyll (GOLD) and in an unrefined form with chlorophyll (RAW). As a reference substance, we used pure CBD (PhytoLab^®^, Vestenbergsgreuth, Germany). Information about the product contents originates from the manufacturers’ specifications. In addition, CannabaOrganics oils have appropriate certificates confirming the concentration of components in each product batch, performed by the accredited EkotechLAB laboratory (Straszyn, Poland). Detailed information about the tested products is presented in Table 5.

The MIC assessment was performed using the microdilution method in Mueller–Hinton broth (MHB) (OXOID, Basingstoke, UK) according to the CLSI guidelines [33], with the addition of 5% ethyl alcohol. The effect of the ethyl alcohol concentration on bacterial growth was assessed in triplicate (Table 6). Each strain was transferred from frozen storage to a Columbia Agar plate (BioMaxima, Lublin, Poland) and incubated for 24 h at 37 °C. The next day, 100 μL of the inoculum (0.5 McF) was transferred to 10 mL of MHB with 5% ethanol (1:100 ratio). All tested products were dissolved in ethyl alcohol to a final cannabinoid concentration of 10 mg/mL. The solutions were further diluted to the following concentrations: 16; 8; 4; 2; 1; 0.5; 0.25; 0.125 [μL/mL]. The evaluation of the effect of alcohol as a solvent on CBD concentrations was conducted by an accredited laboratory. The results will be available by contacting us directly.

Each well contained 100 µL of bacterial suspension and 100 µL of the test product solution. For each bacterial isolate, the MIC was assessed in three replicates. The resazurin sodium salt solution (Acros Organics, Waltham, MA, USA) was used as an indicator of the bacterial metabolism. On each plate, the positive control (bacterial suspension with the addition of MHB solution with 5% ethyl alcohol) and four negative controls (the MHB solution with 5% ethyl alcohol) were included. The polystyrene plates were placed in a humid chamber and incubated for 24 h at 37 °C. The results were determined by measuring the turbidity of the wells according to the EUCAST guidelines [34]. The *E. faecalis* ATCC 29212 strain was used for the quality control.

### 4.4. Statistical Analysis

The statistical analysis was performed using R Statistical Software (v4.4.2; R Core Team 2024). Two independent groups measured on an ordinal scale were compared using the Wilcoxon rank-sum test. Conversely, for dependent ordinal data, the Wilcoxon signed-rank test was used. To assess the effects of multiple predictors and their interaction on ordinal outcomes with multiple categories, an ordinal logistic mixed-effect regression model was fitted using the ordinal package. The model analysis and interpretation were primarily based on the estimated marginal means (EMMs), calculated using the emmeans package. The significance level for all tests was set at α = 0.05. For selected pairwise comparisons following significant main effects or interactions, the Holm correction was applied to control the family-wise error rate.

## 5. Conclusions

In recent years, interest in the potential application of cannabinoids for the treatment of bacterial infections has increased markedly. Preliminary manufacturer-conducted assessments indicate that oils containing cannabidiol (CBD) and other cannabinoids exhibit antibacterial properties. Nevertheless, further comprehensive studies are necessary to elucidate the specific effects of CBD on diverse bacterial species.

Our results demonstrated that the MIC values for all CBD-treated strains were ≤1 μg/mL, confirming their efficacy against the two most frequently isolated nosocomial *Enterococcus* species. Moreover, studies on oils containing various cannabinoids have shown that higher CBD concentrations correlate with stronger antibacterial activity, indicating that CBD is the primary component responsible for this effect. These preliminary investigations provide meaningful insights into the activity of CBD against *Enterococcus* spp. and highlight their potential as a novel antibacterial agent. To date, research has largely neglected interspecies variability, and our findings suggest that exploring these differences may offer a valuable avenue for clarifying the mechanisms underlying CBD’s action in bacterial cells.

## Figures and Tables

**Table 1:** Results of MALDI-TOF MS identification and antibiotic susceptibility determination using the disc diffusion method and the broth microdilution method for *E. faecalis* (*n* = 9) and *E. faecium* (*n* = 11).

No.	Species	SV	Disc-Diffusion Method Results					MIC Values [μg/mL] and Interpretation			
			AMP	IMP	VAN	TEC	LNZ	VAN		TEC	
1.	EFM	2.28	R	R	R	R	S	>16	R	2	S
2.	EFM	2.39	R	R	R	R	S	16	R	1	S
3.	EFM	2.50	R	R	R	S	S	16	R	0.125	S
4.	EFM	2.11	R	R	R	S	S	16	R	0.125	S
5.	EFM	2.27	R	R	R	S	R	>1	R	0.125	S
6.	EFM	2.36	R	R	S	S	S	0.125	S	0.125	S
7.	EFM	2.20	R	R	R	R	S	>16	R	4	R
8.	EFM	2.19	R	R	R	S	R	>16	R	0.25	S
9.	EFM	2.52	R	R	S	S	S	0.125	S	0.125	S
10.	EFM	2.25	R	R	S	S	S	0.125	S	0.125	S
11.	EFM	2.26	R	R	S	S	S	0.125	S	0.125	S
12.	EFA	2.07	S	S	S	S	S	0.125	S	0.125	S
13.	EFA	2.06	S	S	S	S	S	0.5	S	0.125	S
14.	EFA	2.37	S	S	S	S	S	0.125	S	0.125	S
15.	EFA	2.46	R	R	S	S	S	0.25	S	0.125	S
16.	EFA	2.30	S	S	S	S	S	0.125	S	0.125	S
17.	EFA	2.34	S	S	R	R	S	>16	R	>16	R
18.	EFA	2.30	R	R	S	S	S	0.125	S	0.125	S
19.	EFA	2.33	S	S	S	S	R	0.125	S	0.125	S
20.	EFA	2.25	S	S	S	S	R	0.125	S	0.125	S

EFA—*E. faecalis*, EFM—*E. faecium*, AMP—ampicillin, IMP—imipenem, VAN—vancomycin, TEC—teicoplanin, R—resistant, S—susceptible, SV—Score Value.

**Table 2:** Pure CBD (PhytoLab^®^) and CBD crystals 99% (Enecta^®^) MIC values determined for *E. faecalis* (*n* = 9) and *E. faecium* (*n* = 11) strains.

No.	Species	MIC [μg/mL]	
		CBD (PhytoLab^®^)	CBD Crystals 99% (Enecta^®^)
1.	EFM	0.5	0.5
2.	EFM	0.25	0.25
3.	EFM	0.25	0.25
4.	EFM	0.125	0.125
5.	EFM	0.25	0.25
6.	EFM	0.5	0.5
7.	EFM	0.5	0.5
8.	EFM	0.25	0.25
9	EFM	0.25	0.25
10.	EFM	0.5	0.5
11.	EFM	0.25	0.25
12.	EFA	1	1
13.	EFA	1	1
14.	EFA	1	1
15.	EFA	1	1
16.	**EFA**	**1**	**0.5**
17.	EFA	0.5	0.5
18.	EFA	1	1
19.	EFA	1	1
20.	EFA	1	1

CBD—cannabidiol, EFA—*E. faecalis*, EFM—*E. faecium*, MIC—minimum inhibitory concentration. The strain for which the MIC values of both products differed is highlighted in bold.

**Table 3:** Cannabinoid oils (CannabaOrganics^®^) MIC determined for *E. faecalis* (*n* = 9) and *E. faecium* (*n* = 11) strains.

No.	Species	MIC [μg/mL]				
		CBD 20% GOLD	CBD 20% RAW	CBN + CBD 10% RAW	CBG 10% RAW	CBDA + CBD 5% RAW
1.	EFM	0.5	0.25	0.5	4	16
2.	EFM	0.25	0.125	0.5	1	0.25
3.	EFM	0.125	0.125	0.5	0.5	2
4.	EFM	0.125	0.125	0.125	0.125	1
5.	EFM	0.125	0.25	0.5	1	2
6.	EFM	0.5	1	1	4	8
7.	EFM	0.25	0.5	0.5	1	8
8.	EFM	0.25	0.25	0.5	0.5	2
9.	EFM	0.25	0.5	0.125	1	0.25
10.	EFM	0.25	0.5	0.5	2	2
11.	EFA	4	4	>16	8	8
12.	EFM	0.25	0.125	0.5	0.5	0.5
13.	EFA	4	8	>16	16	16
14.	EFA	4	4	>16	8	16
15.	EFA	2	2	>16	4	8
16.	EFA	1	2	8	4	4
17.	EFA	1	0.5	>16	8	8
18.	EFA	2	2	>16	8	8
19.	EFA	4	4	>16	8	16
20.	EFA	2	2	>16	8	8

CBD—cannabidiol, CBDA—cannabidiolic acid, CBG—cannabigerol, CBN—cannabinol, EFA—*E. faecalis*, EFM—*E. faecium*, MIC—minimum inhibitory concentration.

**Table 4:** Origin of *E. faecalis* (*n* = 9) and *E. faecium* (*n* = 11) strains selected for the study.

No.	Species	Clinical Material	Resistance Mechanism
1	EFM	URM	GRE
2	EFM	URM	GRE
3	EFM	URC	VRE
4	EFM	UP	VRE
5	EFM	URC	VRE
6	EFM	URM	-
7	EFM	URM	GRE
8	EFM	URC	VRE, LRE
9	EFM	URM	-
10	EFM	URM	-
11	EFM	URM	-
12	EFA	URM	-
13	EFA	URM	-
14	EFA	URM	-
15	EFA	URN	-
16	EFA	URM	-
17	EFA	URM	GRE
18	EFA	URM	-
19	EFA	URM	LRE
20	EFA	URM	LRE

EFA—*E. faecalis*, EFM—*E. faecium*, UP—ureter prosthesis, URC—urine samples collected through the catheter, URM—midstream urine samples, URN—urine samples collected from a nephrostomy, GRE—Glycopeptide-resistant *Enterococcus*, VRE—Vancomycin-resistant *Enterococcus*, LRE—Linezolid-resistant *Enterococcus.*

**Table 5:** Tested products containing CBD, other cannabinoids, and terpenes.

Product Name	Producer	Form	Concentration of CBD *	Components Other than CBD *
Pure CBD 100%	PhytoLab^®^, Germany	powder	[100.00%]	none [0.00%]
CC 1000 CBD crystals 99%	Enecta^®^, Amsterdam, The Netherlands	powder	[99.00%]	terpenes [1.00%]
CBD 20% GOLD	CannabaOrganics^®^, Warsaw, Poland	oil	>2000 mg/10 mL [90.97%]	CBDA 0.5 mg [0.02%] CBN > 1 mg [0.05%] CBG > 50 mg [2.27%] CBDV > 7 mg [0.32%] CBC > 40 mg [1.82%] terpenes > 100 mg [4.55%]
CBD 20% RAW	CannabaOrganics^®^, Poland	oil	>2000 mg/10 mL [80.32%]	CBDA > 250 mg [10.04%] CBN > 3 mg [0.12%] CBG > 100 mg [4.02%] CBDV > 7 mg [0.28%] CBC > 30 mg [1.20%] terpenes > 100 mg [4.2%]
CBN + CBD 10% RAW	CannabaOrganics^®^, Poland	oil	>500 mg/10 mL [40.34%]	CBN > 500 mg [40.34%] CBDA > 1 mg [0.08%] CBDV > 3 mg [0.24%] CBG > 110 mg [8.87%] CBGA > 0.5 mg [0.04%] CBC > 25 mg [2.02%] terpenes > 100 mg [8.07%]
CBDA + CBD 5% RAW	CannabaOrganics^®^, Poland	oil	>330/10 mL [46.74%]	CBDA > 170 mg [24.08%] CBDV > 2 mg [0.28%] CBG > 19 mg [2.70%] CBGA > 8 mg [1.13%] CBN > 1 mg [0.14%] CBC > 24 mg [3.40%] CBL > 2 mg [0.28%] terpenes > 150 mg [21.25%]
CBG 10% RAW	CannabaOrganics^®^, Poland	oil	150 mg/10 mL [11.45%]	CBG > 1000 mg [76.34%] CBGA > 2 mg [0.15%] CBDA > 1 mg [0.08%] CBDV > 3 mg [0.23%] CBN > 1 mg [0.08%] CBC > 50 mg [3.81%] CBL > 3 mg [0.23%] terpenes > 100 mg [7.63%]

* According to the producer specifications of the product; CBC—cannabichromene, CBD—cannabidiol, CBDA—cannabidiolic acid, CBDV—cannabidivarine, CBG—cannabigerol, CBGA—cannabigerol acid, CBL—cannabicyclol, CBN—cannabinol.

**Table 6:** Assessment of *E. faecalis* (*n* = 9) and *E. faecium* (*n* = 11) growth in MHB with the addition of different ethyl alcohol concentrations.

No.	Species	Growth in MHB with Ethyl Alcohol Concentration									
		1%	2%	3%	4%	5%	6%	7%	8%	9%	10%
1.	EFM	yes	yes	yes	yes	**yes**	yes	no	no	no	no
2.	EFM	yes	yes	yes	yes	**yes**	no	no	no	no	no
3.	EFM	yes	yes	yes	yes	**yes**	yes	no	no	no	no
4.	EFM	yes	yes	yes	yes	**yes**	yes	yes	no	no	no
5.	EFM	yes	yes	yes	yes	**yes**	yes	yes	no	no	no
6.	EFM	yes	yes	yes	yes	**yes**	yes	yes	no	no	no
7.	EFM	yes	yes	yes	yes	**yes**	yes	yes	no	no	no
8.	EFM	yes	yes	yes	yes	**yes**	yes	yes	yes	no	no
9	EFM	yes	yes	yes	yes	**yes**	yes	no	no	no	no
10.	EFM	yes	yes	yes	yes	**yes**	yes	yes	no	no	no
11.	EFM	yes	yes	yes	yes	**yes**	yes	yes	no	no	no
12.	EFA	yes	yes	yes	yes	**yes**	yes	yes	no	no	no
13.	EFA	yes	yes	yes	yes	**yes**	yes	no	no	no	no
14.	EFA	yes	yes	yes	yes	**yes**	yes	no	no	no	no
15.	EFA	yes	yes	yes	yes	**yes**	yes	yes	no	no	no
16.	EFA	yes	yes	yes	yes	**yes**	yes	yes	no	no	no
17.	EFA	yes	yes	yes	yes	**yes**	no	no	no	no	no
18.	EFA	yes	yes	yes	yes	**yes**	yes	no	no	no	no
19.	EFA	yes	yes	yes	yes	**yes**	yes	yes	no	no	no
20.	EFA	yes	yes	yes	yes	**yes**	yes	no	no	no	no

EFA—*E. faecalis*, EFM—*E. faecium*, MHB—Mueller–Hinton Broth. The highest concentration of ethyl alcohol that supported the growth of all tested isolates is indicated in bold.

## Data Availability

Raw data will be made available upon request to the corresponding author.

## Abbreviations

The following abbreviations are used in this manuscript:

ART	aligned rank transform
ATCC	American Type Culture Collection
CBC	cannabichromene
CBD	cannabidiol
CBDA	cannabidiolic acid
CBDV	cannabidivarine
CBG	cannabigerol
CBGA	cannabigerol acid
CBL	cannabicyclol
CBN	cannabinol
CLSI	Clinical and Laboratory Standards Institute
ECDC	European Centre for Disease Prevention and Control
ECDD	Expert Committee on Drug Dependence
EUCAST	European Committee for Microbial Susceptibility Testing
GRE	glycopeptide-resistant *Enterococcus*
ICU	intensive care units
LRE	linezolid-resistant *Enterococcus*
MALDI-TOF MS	matrix assisted laser desorption/ionization—time of flight mass spectrometry
MDR	multi drug resistant
MHA	Mueller–Hinton agar
MHB	Mueller–Hinton broth
MIC	minimal inhibitory concentration
MRSA	methicillin-resistant *Staphylococcus aureus*
PAI	pathogenicity islands
SSI	surgical site infections
SV	Score Value
UTI	urinary tract infections
VRE	vancomycin-resistant *Enterococcus*
